# Influenza A virus infection of vascular endothelial cells induces GSK-3β-mediated β-catenin degradation in adherens junctions, with a resultant increase in membrane permeability

**DOI:** 10.1007/s00705-014-2270-5

**Published:** 2014-11-12

**Authors:** M. Hiyoshi, I. L. Indalao, M. Yano, K. Yamane, E. Takahashi, H. Kido

**Affiliations:** 1 Division of Enzyme Chemistry, Institute for Enzyme Research, The University of Tokushima, Kuramoto-cho 3-18-15, Tokushima, 770-8503 Japan; 2 Present Address: Department of Biochemistry, Shimane University Faculty of Medicine, Izumo, Shimane 693-8501 Japan; 3 Present Address: Department of Nutrition, School of Human Cultures, The University of Shiga Prefecture, Hikone, Shiga 522-8533 Japan

## Abstract

Multiorgan failure with vascular hyperpermeability is the final outcome in the progression of seasonal influenza virus pneumonia and influenza-associated encephalopathy, and it is also common in infection with highly pathogenic avian influenza virus. However, the precise molecular mechanism by which influenza virus infection causes vascular endothelial cell hyperpermeability remains poorly defined. We investigated the mechanisms of hyperpermeability of human umbilical vein endothelial cells infected with influenza A virus (IAV)/Puerto Rico/8/34 (PR8) (H1N1). The levels of β-catenin, a key regulatory component of the vascular endothelial-cadherin cell adhesion complex, were markedly decreased during infection for 28 h, with increments of vascular hyperpermeability measured by transendothelial electrical resistance. Lactacystin (at 2 μM), a proteasome inhibitor, inhibited the decrease in β-catenin levels. Since the N-terminal phosphorylation of β-catenin by glycogen synthase kinase (GSK)-3β is the initiation step of proteasome-dependent degradation, we examined the effects of GSK-3β suppression by RNA interference in endothelial cells. IAV-infection-induced β-catenin degradation was significantly inhibited in GSK-3β-knockdown cells, and transfection of cells with recombinant β-catenin significantly suppressed IAV-induced hyperpermeability. These findings suggest that IAV infection induces GSK-3β-mediated β-catenin degradation in the adherens junctional complexes and induces vascular hyperpermeability. The *in vitro* findings of β-catenin degradation and activation of GSK-3β after IAV infection were confirmed in lungs of mice infected with IAV PR8 during the course of infection from day 0 to day 6. These results suggest that GSK-3β-mediated β-catenin degradation in adherens junctions is one of the key mechanisms of vascular hyperpermeability in severe influenza.

## Introduction

Influenza A virus (IAV) is the most common infectious pathogen in humans and causes significant morbidity and mortality, particularly in infants and the elderly population [1, 2]. Multiorgan failure (MOF) with vascular hyperpermeability is reported in the progressive stage of seasonal influenza virus pneumonia, particularly in patients with underlying risk factors [3], and is also common in infections caused by highly pathogenic avian influenza virus [4]. Vascular hyperpermeability caused by destruction of a tight junction constituent, zonula occludens-1 (ZO)-1, of the blood-brain-barrier (BBB) in brain endothelial cells is also reported in influenza-associated encephalopathy in infancy and early childhood in East Asians [5–8]. We reported previously that the “influenza–cytokine–trypsin and matrix metalloprotease-9” cycle is a key pathogenic mechanism in the interaction between IAV infection and vascular endothelial cells and their hyperpermeability in severe influenza [8, 9].

The state of hypercytokinemia in IAV infection (e.g., high levels of tumor necrosis factor, interleukin 6, and interleukin-1β), called a “cytokine storm”, affects cell adhesion, permeability, apoptosis, and mitochondrial energy metabolism and reactive oxygen species, potentially resulting in vascular dysfunction, hyperpermeability and MOF [10, 11], although their precise mechanisms have not been elucidated so far. Furthermore, IAV infection induces upregulation of trypsin and matrix metalloprotease-9 through an “influenza–cytokine–protease” cycle in various organs and vascular endothelial cells [8, 9, 11, 12], and an increase in the levels of these proteases in the extracellular space caused by this cycle results in the degradation of vascular basement membranes and the extracellular matrix, resulting in vascular hyperpermeability and inflammatory cell migration. Trypsin promotes viral entry and replication because IAV does not encode a viral hemagglutinin processing protease in its own genes, and post-transcriptional hemagglutinin cleavage by cellular trypsin-type proteases is needed for membrane fusion activity, virus entry into cells, and multiple cycles of viral replication [13–15]. In addition, trypsin evokes cytokine release via the proteinase-activated receptor (PAR)-2 [16] and also plays a role in BBB destruction by increasing intracellular calcium concentrations ([Ca^2+^]_i_) and loss of tight-junction protein ZO-1 via PAR-2 in the BBB [8, 17].

For the control of vascular permeability, there is another major type of cell-to-cell junction, the adherens junction, which is ubiquitously distributed along the vascular tree and is expressed in both blood and lymphatic vessels [18]. The adherens junction is formed by the transmembrane adhesion molecule vascular endothelial (VE)-cadherin and its cytoplasmic tail binding molecules, β-catenin and plakoglobin, which anchor to actin via α-catenin and stabilize the junction [19].

In this study, we investigated the mechanisms of IAV-induced disruption of adherens junctions and vascular hyperpermeability in human endothelial cells *in vitro* and confirmed these findings *in vivo*. We found that GSK-3β-mediated β-catenin degradation in the VE-cadherin complex in the adherens junction of human endothelial cells was associated with increased hyperpermeability after IAV infection.

## Materials and methods

### Cell culture

Normal human umbilical vein endothelial cells (HUVECs) (Kurabo Industries, Osaka, Japan) on type-I-collagen-coated dishes (10 cm diameter) were grown in HuMedia-EG2 culture medium using the protocol supplied by the manufacturer. The cells were cultured in a humidified atmosphere of 5 % CO_2_–95 % air gas mixture at 37 °C.

### Influenza A virus infection

IAV/PR/8/34 (H1N1) was provided by the Research Foundation for Microbial Diseases of Osaka University (Kagawa, Japan). Before infection of HUVEC with IAV PR8, the culture medium was replaced with serum-free HuMedia-EB2 infection medium containing 0.1 % bovine serum albumin (BSA). The cells were infected with IAV PR8 at a multiplicity of infection (MOI) of 1. After incubation for 1 h, the infection medium was replaced with a culture medium containing various reagents, and the cells were incubated for another 28 h.

For animal experiments, specified-pathogen-free 4-week-old weanling C57BL/6CrSlc female mice were obtained from Japan SLC and maintained in a 12-h light/dark cycle in a temperature-controlled room with free access to food and water. All animals were treated according to the Guide for the Care and Use of Laboratory Animals (NIH Publication No. 85-23, 1996), and the study was approved by the Animal Care Committee of the University of Tokushima. Under ketamine and xylazine anesthesia, 100 plaque-forming units of IAV PR8 in 20 μL of saline or saline alone as an uninfected control was instilled intranasally in mice. Body weight and survival were monitored daily, and the animals were assessed visually for signs of clinical disease including inactivity, ruffled fur, labored respiration and huddling behavior. Mice that lost ≥30 % of their original body weight and/or displayed evidence of pneumonia were sacrificed by overdose of intraperitoneal ketamine and xylazine. These experiments were conducted under animal BSL2 conditions.

### Western immunoblotting

HUVECs after various treatments were lysed in radioimmunoprecipitation assay (RIPA) buffer (50 mM Tris-HCl, pH 8.0, 150 mM NaCl, 10 % glycerol, 1 % NP 40, 0.5 % deoxycholate, 0.4 mM EDTA, and 0.5 mM sodium orthovanadate) for 30 min at 4 °C. Mouse lungs isolated during the course of IAV infection (from day 0 to day 6) were homogenized with five volumes of RIPA buffer. Cell lysate and lung homogenate were centrifugation at 10,000×*g* for 30 min. The prepared cell lysates (20 μg protein/lane) and lung extracts (20 μg protein/lane) were separated by sodium dodecyl sulfate polyacrylamide gel electrophoresis (SDS-PAGE) using 10-to-20 % gradient gel and transferred onto an Immobilon-P membrane (Millipore, Bedford, MA). After blocking with 5 % non-fat milk in 20 mM Tris-HCl, pH 7.5, 150 mM NaCl and 0.05 % Tween 20, the membranes were incubated with optimal primary antibodies for 2 h. The primary antibodies included anti-β-catenin, anti-VE-cadherin, anti-β-actin and anti-glycogen synthase kinase (GSK)-3β (all from Santa Cruz Biotechnology, Santa Cruz, CA), anti-phospho-GSK-3β (Ser9) (Cell Signaling Technology, Beverly, MA) and anti-IAV (Takara). Thereafter, peroxidase-conjugated anti-mouse (Invitrogen, Carlsbad, CA), -rabbit (Sigma, St. Louis, MO) or -goat (Sigma) IgG antibodies were incubated for 30 min, followed by enhanced chemiluminescence reagents (GE Healthcare Biosciences, Uppsala, Sweden) for 1 min.

### Reverse transcription polymerase chain reaction (RT-PCR)

Total RNA was extracted from HUVECs using an RNeasy Mini Kit (QIAGEN, Hilden, Germany). RT-PCR was carried out using a One-Step RT-PCR Kit (QIAGEN) according to the instructions provided by the manufacturer. To amplify the PCR product of β-catenin, the following primer pair was used (forward primer, 5′-TTTGGCTGAACCATCACAGA-3′; and reverse primer, 5′-TGTTGAGCAAGGCAACCATT-3′). The products were examined by agarose gel electrophoresis after 25 cycles.

### Relative quantitation analysis

Protein or mRNA bands detected on X-ray films or pictures were calculated by densitometric analysis using Just TLC (Sweday, Lund, Sweden). The relative levels of the immunoblot or RT-PCR bands were expressed as mean values ±SD.

### RNA interference

The sequence of the sense strand used to generate specific siRNA was obtained as follows: GSK-3β, NCBI Reference Sequence: NG_012922.1, 5′-AAATCTCTTGTCCTGCAATAC-3′. A small interfering RNA (siRNA) was synthesized using a Silencer siRNA Construction Kit (Ambion, Austin, TX) according to the instructions supplied by the manufacturer. HUVECs were transfected with the siRNA at 10 nM, using Oligofectamine Transfection Reagent (Invitrogen) and grown for 72 h to allow sufficient decrease in the expression of this molecule.

### Assessment of hyperpermeability by trans-endothelial electrical resistance (TEER) and transfection with recombinant β-catenin

HUVEC were plated in the upper chamber of type I collagen-coated cell culture inserts with a membrane of 3.0 μm pore size. After infection of the cells with IAV PR8 at a MOI of 1 for 24 h, some of the cells on the plates were transfected with recombinant β-catenin (1.2 μg/mL) (Millipore). Recombinant β-catenin was exposed to the transfection reagent TransIT-LT1 polyamine (Mirus, Madison, WI) for 15 min before transfection. After transfection for 4 h, the TEER across monolayers of endothelial cells was measured using Millicell-ERS (Millipore) using the method described previously [20, 21]. The electrical resistance of blank inserts containing the medium only was subtracted from the TEER measurements made from inserts containing confluent endothelial cell monolayers.

### Statistical analysis

The mean values ±SD of individual groups were calculated, and the differences between groups were analyzed using the unpaired *t*-test or multiple comparisons (Bonferroni or Dunnett tests) after one-way analysis of variance (ANOVA). All statistical analyses were performed using Microsoft Excel (Microsoft, Redmond, WA) add-in Ekuseru-Toukei 2010 version 1.10 (Social Survey Research Information Co.). All values are two-tailed and those less than 0.05 were considered statistically significant.

## Results

### IAV infection downregulates β-catenin significantly, but not VE-cadherin, in adherens junctional proteins in HUVECs

**Fig. 1:**
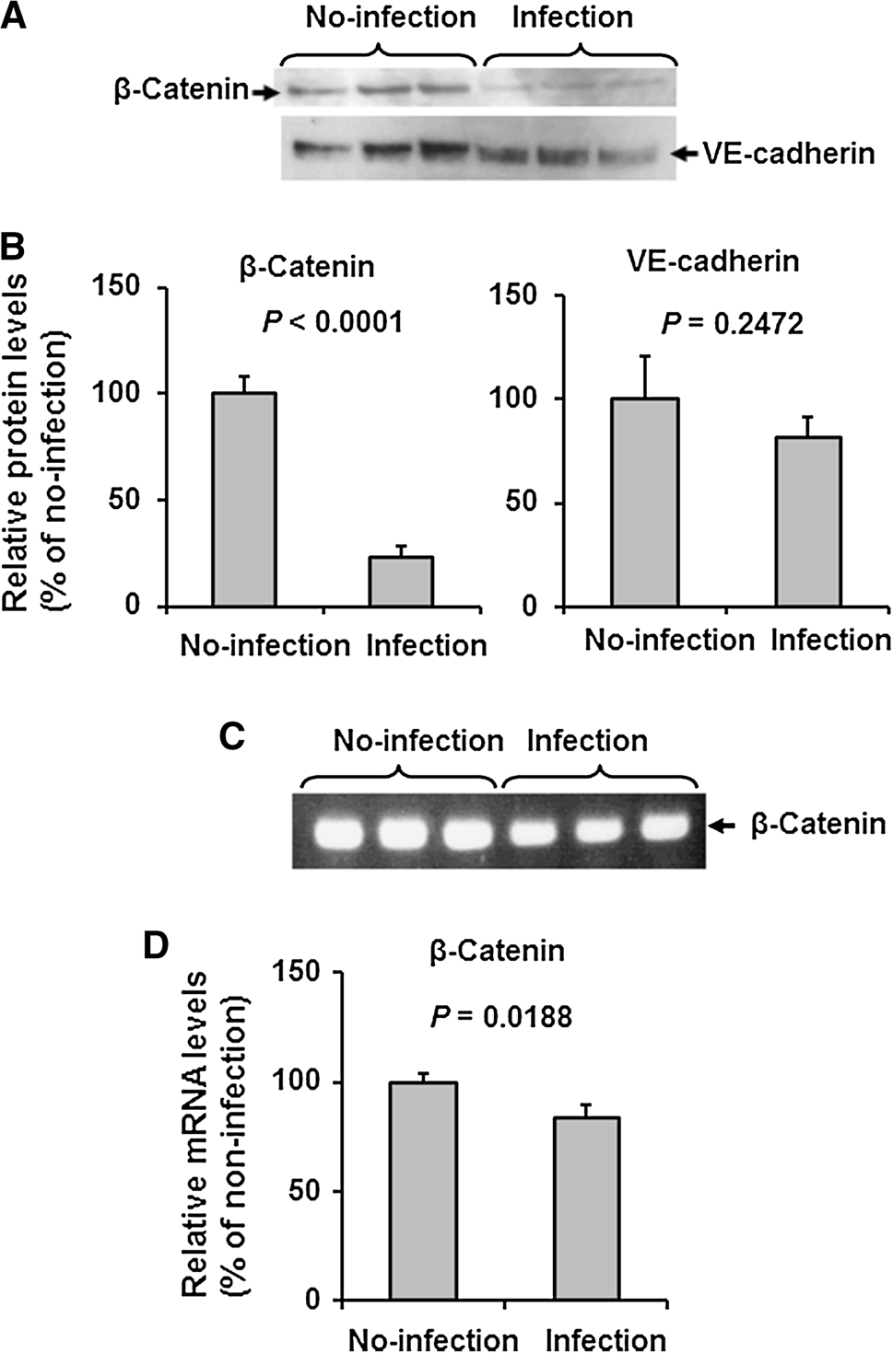
Infection with IAV reduces the expression of β-catenin in adherens junctional proteins in HUVECs. **A.** Representative immunoblot (from three separate experiments) of β-catenin and VE-cadherin in cell lysates (20 μg protein/lane) of HUVECs after infection for 28 h with or without IAV PR8. As a control, saline was used as no-infection agent. **B.** Relative levels of β-catenin and VE-cadherin in the blot in panel A (n = 3), determined as described in Materials and methods. Statistical analysis was performed using the unpaired *t*-test. **C.** Representative RT-PCR data of β-catenin in control (uninfected) and IAV-infected HUVECs at 28 h post-transfection. D: Relative levels of β-catenin in the RT-PCR experiment shown in panel C (n = 3). Statistical analyses were conducted using the unpaired *t*-test

Figures 1A and B show the results of immunoblotting analyses of β-catenin and VE-cadherin, the major adherens junctional proteins, in HUVECs infected with IAV PR8 at an MOI of 1 for 28 h or in uninfected cells. Infection significantly suppressed the expression of β-catenin to 24 % of the no-infection control (*P* < 0.0001). The expression of the transmembrane adhesive component VE-cadherin also tended to decrease, though not significantly, after infection (*P* = 0.2472). Quantitative RT-PCR analysis showed a slight, but significant, decrease in the β-catenin mRNA level to 84 % of the no-infection control (*P* = 0.0188) in infected HUVECs (Fig. 1C and D). The discrepancy between the suppression levels of β-catenin protein and those of β-catenin mRNA after IAV infection indicates that the marked suppression of the β-catenin protein level was probably due to enhanced β-catenin protein degradation.

### Lactacystin inhibits IAV-induced β-catenin degradation

**Fig. 2:**
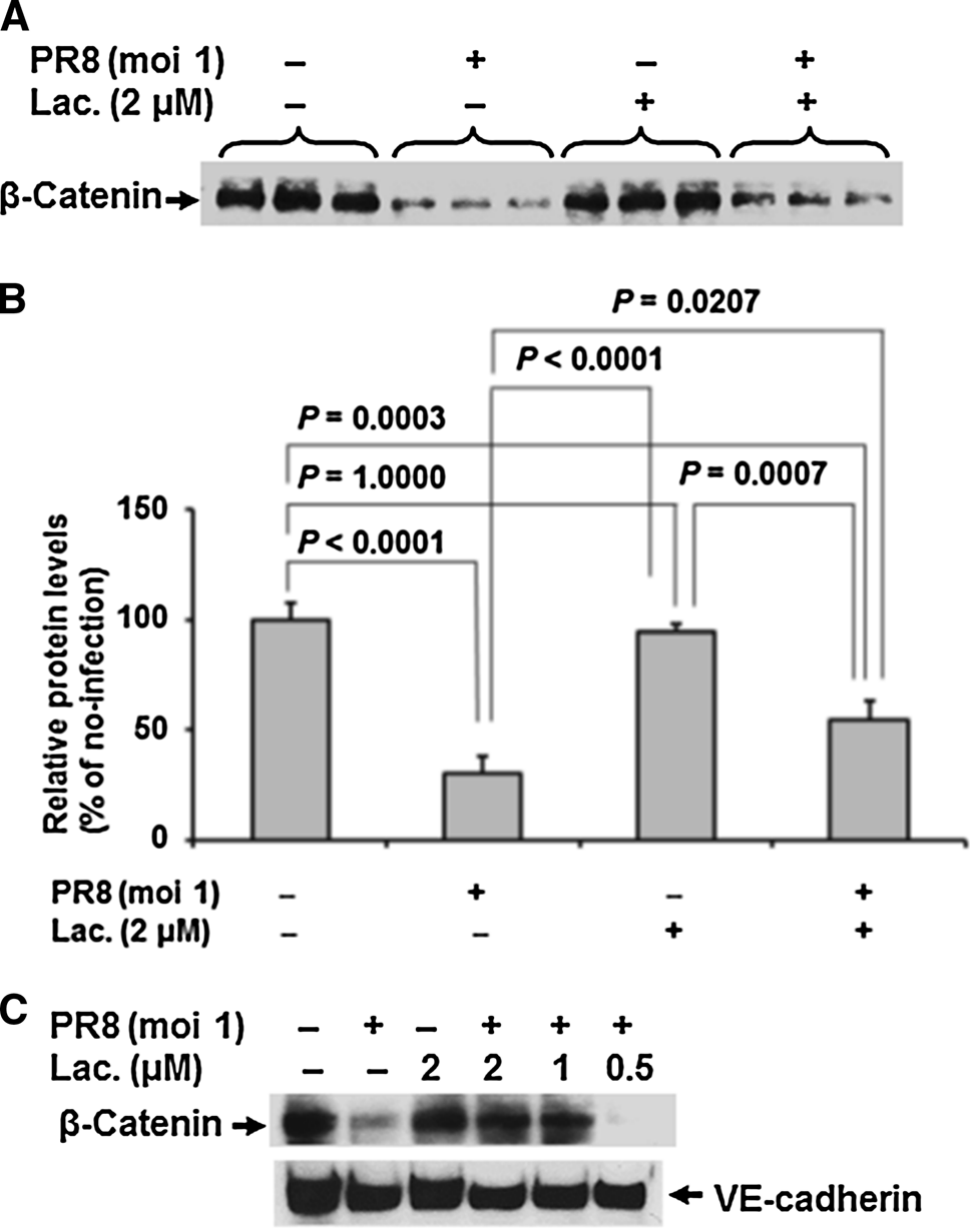
Lactacystin inhibits IAV-induced β-catenin suppression. **A.** Representative immunoblot (from three separate experiments) of β-catenin in cell lysates (20 μg protein/lane) of control (uninfected) or IAV-infected HUVECs treated with or without lactacystin for 28 h. **B.** Relative level of β-catenin in the blot in panel A (n = 3). Multiple comparisons tests (Bonferroni test after one-way analysis of variance (ANOVA) were used for statistical analysis. **C.** Dose-response effect of lactacystin on β-catenin and VE-cadherin expression. HUVECs were treated with various concentrations of lactacystin (from 0 to 2 μM) at the time of infection and then incubated for 28 h. A representative immunoblot of β-catenin and VE-cadherin in cell lysates (20 μg protein/lane) with or without infection is shown

To verify the above-stated findings, HUVECs were treated with 2 μM lactacystin (Sigma), a proteasome inhibitor [22], at the time of PR8 infection and the cells were incubated for 28 h. Under no-infection conditions, lactacystin did not affect β-catenin protein levels in HUVEC (*P* = 1.0000) (Fig. 2A and B). In contrast, lactacystin significantly abrogated the suppression of the β-catenin protein level in IAV PR8-infected HUVECs (*P* = 0.0207), and the levels were 1.8-fold higher than those in infected cells that were not treated with lactacystin. The inhibitory effect of lactacystin was dose-dependent, and lactacystin at 0.5 μM was not enough to restore the β-catenin levels (Fig. 2C). Expression of VE-cadherin was not affected by treatment with various doses of lactacystin. These results confirmed that the downregulation of the β-catenin protein level in IAV PR8-infected HUVECs is the result of enhanced proteasomal degradation.

### IAV PR8 infection activates GSK-3β in HUVECs

**Fig. 3:**
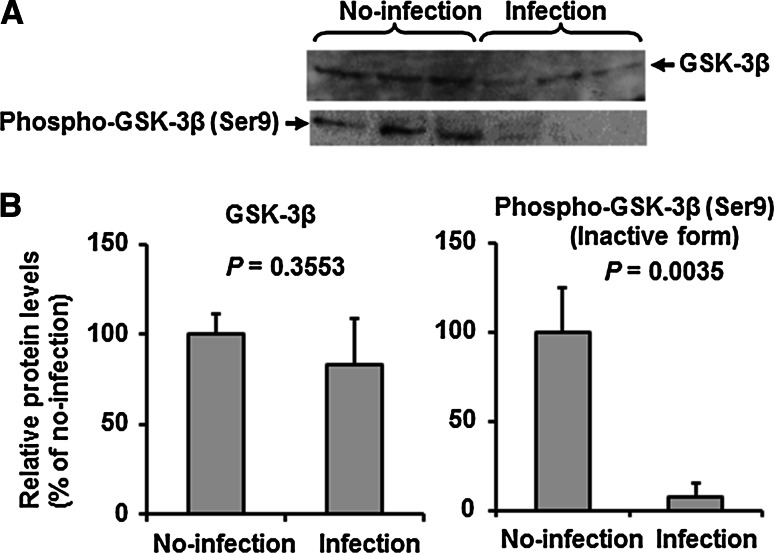
IAV infection induces suppression of phosphorylated GSK-3β in HUVECs. **A.** Representative immunoblots (from three separate experiments) of total GSK-3β and phosphor-GSK-3β (Ser9) in cell lysates (20 μg protein/lane) of control (uninfected) or IAV-infected HUVECs. **B.** Relative levels of total GSK-3β and phosphor-GSK-3β (Ser9) in the blot in panel A (n = 3). Data are mean ± SD. Statistical analyses were conducted using the unpaired *t*-test

β-Catenin, which is phosphorylated by GSK-3β at residues 37 and 33, is recognized by the β-TrCP E3-ligase complex, ubiquitylated, and rapidly degraded by the 26S proteasome [23, 24]. To elucidate the mechanism(s) of enhanced β-catenin degradation in HUVEC by IAV infection, the expression level of the active form of dephosphorylated GSK-3β (Ser9) was analyzed by western immunoblotting using specific antibodies against GSK-3β and phospho-GSK-3β (Ser9) to detect the inactive form of phosphorylated GSK-3β (Fig. 3A and B). The total amount of GSK-3β in IAV PR8-infected cells was only slightly decreased to 83 % of the no-infection control cells, and the decrease was not significant (*P* = 0.3553). In contrast, IAV infection markedly decreased the level of the phosphorylated-GSK-3β (Ser9) form to 8 % (*P* = 0.0035). These results indicate that IAV infection induces GSK-3β in HUVECs from the inactive phosphorylated-GSK-3β (Ser9) form to the active dephosphorylated-GSK-3β (Ser9) form.

### GSK-3β knockdown protects against IAV infection-induced suppression of β-catenin expression

**Fig. 4:**
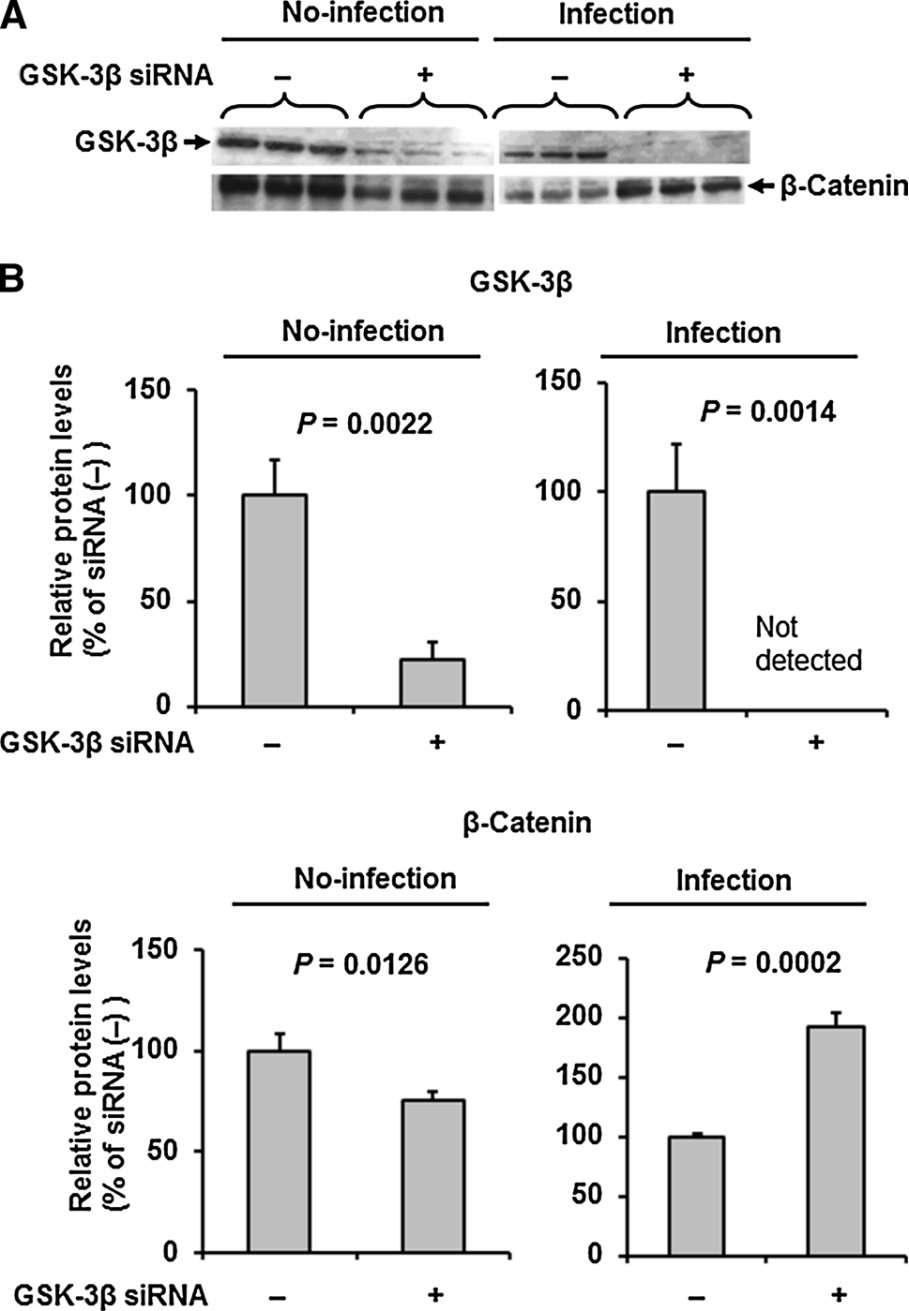
GSK-3β knockdown protects against IAV-induced suppression of β-catenin. **A.** Representative immunoblots (from three separate experiments) of GSK-3β and β-catenin in cell lysates (20 μg protein/lane) of HUVECs pre-treated with or without GSK-3β knockdown under control (no-infection) and IAV-infection conditions. **B.** Relative levels of GSK-3β and β-catenin in the bolt in panel A (n = 3). Differences between groups were analyzed using the unpaired *t*-test

Since degradation of β-catenin, a major component of the VE-cadherin complex, is initiated by the activation of GSK-3β, we next analyzed the effects of GSK-3β gene-silencing on the level of β-catenin in IAV-infected and uninfected HUVECs (Fig. 4A and B). HUVECs treated with or without GSK-3β knockdown were infected with IAV PR8 at a MOI of 1 and β-catenin levels in the cells were measured by western immunoblotting at 28 h postinfection. Under no-infection conditions, GSK-3β knockdown treatment resulted in suppression of GSK-3β expression in HUVEC to about 20 % of the level without treatment. Although GSK-3β expression was downregulated in HUVECs by IAV infection, the expression was further suppressed by GSK-3β gene silencing to an undetectable level. The β-catenin expression level in HUVECs treated with GSK-3β knockdown under no-infection conditions was 76 % of those without knockdown treatment (*P* = 0.0126). However, the β-catenin expression level in GSK-3β knockdown cells was 1.9-fold of that in HUVECs without treatment under infection conditions (*P* = 0.0002). These data suggest that the β-catenin level in the VE-cadherin complex is strictly regulated by GSK-3β in IAV-infected HUVECs.

### Transfection with β-catenin restores the IAV-infection-disrupted barrier function

**Fig. 5:**
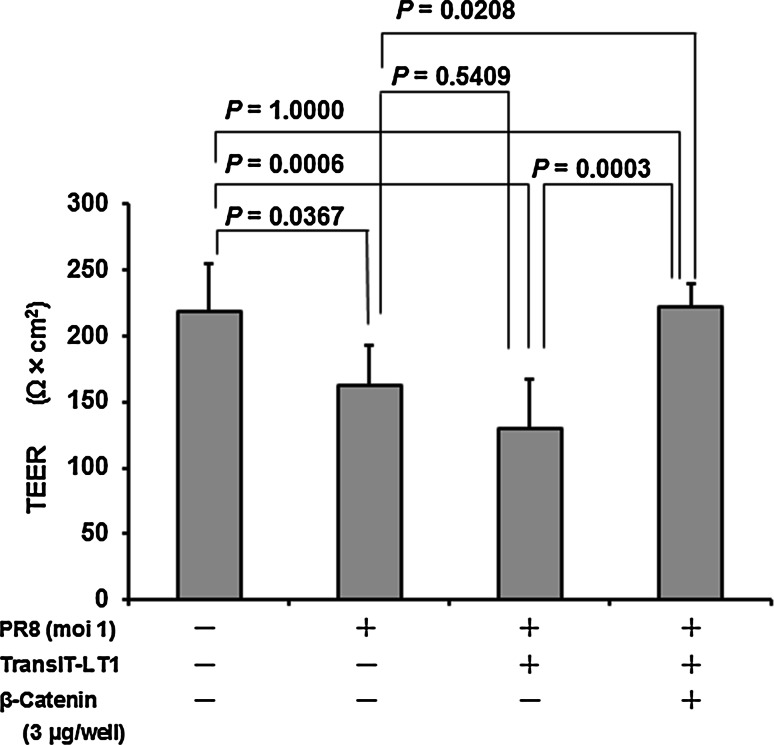
Transfection with recombinant β-catenin abrogates IAV infection-induced impairment of barrier function. TEER data across monolayers of control (uninfected) and IAV-infected HUVECs (n = 6), and with or without recombinant β-catenin transfection were analyzed using multiple comparison (Bonferroni test) after one-way analysis of variance (ANOVA)

The hyperpermeability of monolayers of HUVECs was measured by TEER. Infection of HUVECs with IAV PR8 at an MOI of 1 for 24 h resulted in a significant decrease in TEER (*P* = 0.0367), indicating cell hyperpermeability (Fig. 5). Transfection of the vehicle (TransIT-LT1 polyamine) tended to reduce TEER, albeit insignificantly (*P* = 0.5409). However, pretreatment of HUVECs by transfection with recombinant β-catenin for 4 h markedly increased TEER (*P* = 0.0003), and the monolayer permeability was almost completely restored to the levels recorded in uninfected and untransfected cells.

### Changes in expression levels of adherens junctional proteins and GSK-3β in lungs of mice infected with IAV PR8

**Fig. 6:**
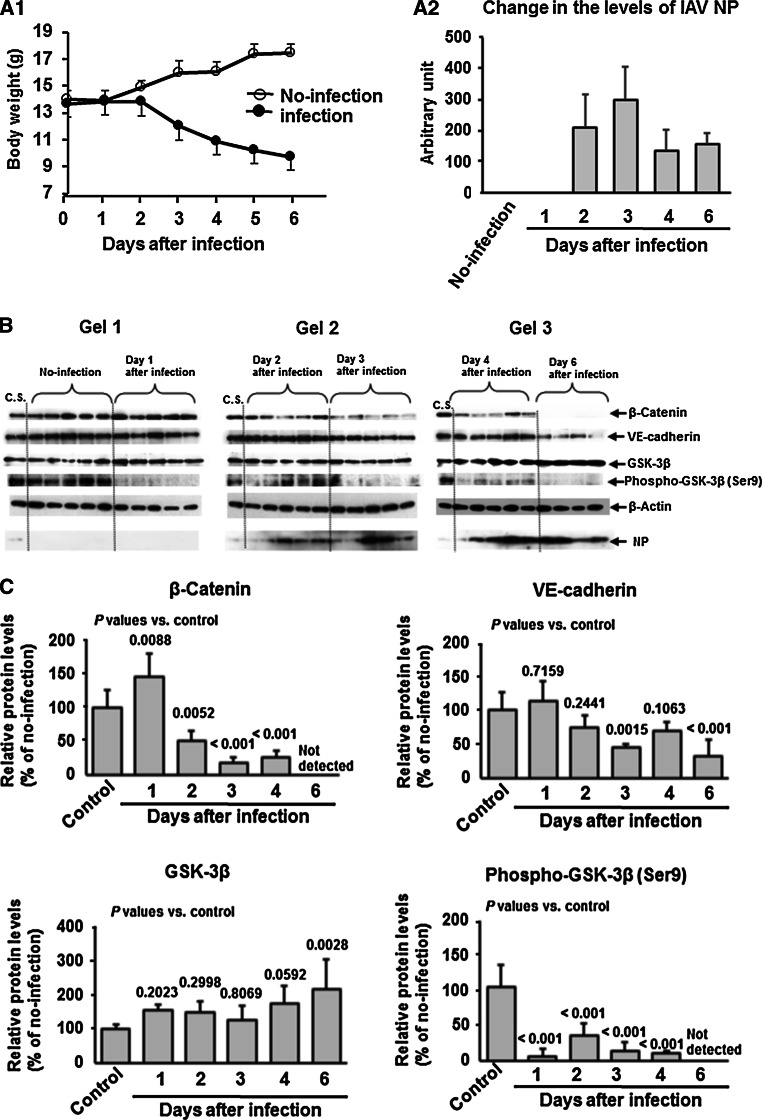
β-Catenin suppression and GSK-3β activation in lungs of IAV-infected mice. A1, change in body weight of infected and uninfected mice (five mice in each group). A2, change in viral NP levels in the lungs monitored by western immunoblotting (B). One infected animal died on day 6 postinfection. The values represent the mean ± SD of five or four mice. B and C, Data represent the expression levels (B) and relative quantified data (C) of β-catenin, VE-cadherin, GSK-3β, and phospho-GSK-3β (Ser9) in lung extracts (20 μg protein/lane) of control (no-infection) and IAV-infected mice from day 1 to day 6 postinfection. C.S., calibration standard (mixture of uninfected sample except for NP analysis) to normalize the intensity of protein bands in different gels. As the C.S. for NP, a sample from one animal after infection for 4 days was used. β-Actin was used as an internal control. Data are representative of three separate experiments. Multiple comparison (Dunnett test) after ANOVA were used for statistical analysis. *P*-values are relative to the control (no infection)

The time courses of change in β-catenin, VE-cadherin, GSK-3β, phospho-GSK-3β (Ser 9) and viral nucleoprotein (NP) expression levels were analyzed in the lungs of mice infected intranasally with IAV PR8 at 100 PFU from day 0 to day 6. The average body weight of infected mice started to fall at day 3 postinfection and an approximately 26 % loss relative to the original weight was observed on day 6, but the average body weight of uninfected mice showed a continuous increase (Fig. 6A1). All animals survived during the experimental period except one infected animal, which died on day 6.

The viral NP levels in the lungs monitored by western immunoblotting showed that they started to increase at day 2 postinfection, reaching a peak at day 3 and then gradually decreasing (Fig. 6A2). Progressive and marked downregulation of β-catenin was noted during the infection period from day 2 to day 6 (Fig. 6B and C). This was coupled with simultaneous downregulation of phosphor-GSK-3β (Ser 9) (inactive form) expression in the lungs, and marked downregulation began at day 1 postinfection and persisted until day 6 postinfection. In contrast to these changes, total GSK-3β expression was steady until day 4 postinfection and was significantly upregulated at day 6. A mild and continuous decrease in the expression of VE-cadherin was detected until day 3 post-expression, followed by a marked decrease in expression from day 4 to day 6 postinfection. Changes in these expression levels were observed in the lungs at day 6 postinfection, the starting point of an obvious multi-organ failure phase with vascular hyperpermeability [15]. These *in vivo* changes in lung tissue added support to the *in vitro* findings of increased hyperpermeability after infection of HUVECs with IAV PR8.

## Discussion

The present study resulted in several new findings: (i) IAV PR8 infection markedly decreased the level of a regulatory component of the VE-cadherin cell adhesion complex, β-catenin, in association with enhanced permeability of human vascular endothelial cells. (ii) The decrease in β-catenin protein levels in the cells was the result of degradation by activation of GSK-3β, and the degradation was inhibited by lactacystin, a proteasome inhibitor. (iii) IAV infection-induced β-catenin degradation was suppressed in HUVECs by GSK-3β knockdown and restoration of cellular barrier function by transfection with β-catenin. (iv) There was marked downregulation of adherens junctional protein β-catenin and upregulation of dephosphorylated GSK-3β (active GSK-3β) in the lungs of mice infected with IAV PR8.

Until recently, anti-influenza agents that inhibit viral replication have represented the best treatment option. However, MOF appears just after influenza virus peak proliferation with the initiation of host immune responses and development of metabolic disorders [11, 15], and thus the use of antiviral neuraminidase inhibitors is not suitable for the treatment of MOF after viral proliferation. MOF induced by severe IAV infection is the final outcome of circulatory failure, hypoxemia, and vascular hyperpermeability of endothelial cells through the “influenza–cytokine–trypsin and matrix metalloprotease-9” cycle [8, 11], with disruption of vascular junctions. In a series of studies, we reported previously that upregulation of trypsin in brain vascular endothelial cells by pro-inflammatory cytokines after IAV infection plays a pivotal role in the destruction of tight junctions in the BBB through an increase in [Ca^2+^]_i_ and loss of ZO-1 via proteinase-activated receptor-2 signaling [8, 17].

Adherens junction, another major type of junction in the vascular endothelium, plays important roles in contact inhibition of endothelial cell growth and paracellular permeability to circulating leukocytes and solutes. Adherens junctions are ubiquitously distributed, and endothelial cells in all types of blood vessels express VE-cadherin. The cytoplasmic tail of VE-cadherin binds β-catenin and plakoglobin at the carboxy-terminal region, both of which link α-catenin and anchor the complex to the actin cytoskeleton [18]. The first of these, β-catenin is a major regulatory component of the VE-cadherin-β-catenin adhesive complex, and its level is tightly regulated by GSK-3β, followed by the phosphorylation-dependent ubiquitin-proteasome degradation pathway in a manner similar to the phosphorylation-dependent degradation of IκB [23].

As an extension to our previous studies on the mechanisms of vascular hyperpermeability in MOF caused by severe IAV infection, we studied the mechanisms of destruction of the VE-cadherin-β-catenin adhesive complex and vascular hyperpermeability after IAV infection in human endothelial cells, both *in vitro* and *in vivo*. Our results showed marked β-catenin degradation by proteasomes and a significant decrease in phosphorylated GSK-3β (Ser9) expression in HUVECs after IAV infection (Figs. 1, 3 and 6). The findings of downregulation of β-catenin after IAV infection through the activation of GSK-3β followed by the phosphorylation-dependent ubiquitin-proteasome degradation pathway were supported by the experiments of GSK-3β gene silencing. GSK-3β-gene-silenced HUVECs showed almost complete protection against β-catenin downregulation in cells infected with IAV (Fig. 4), and β-catenin degradation was inhibited by lactacystin (Fig. 2). In addition, expression of recombinant β-catenin in HUVECs almost completely restored monolayer hyperpermeability induced by IAV infection. These results indicate that the level of β-catenin in the VE-cadherin-β-catenin adhesive complex is important for regulation of vascular permeability and that the β-catenin level is regulated by the GSK-3β-mediated β-catenin degradation pathway. The findings with IAV-infected HUVECs support previous studies on β-catenin degradation in the absence of Wingless/Wnt signaling pathway via GSK-3β-mediated β-catenin phosphorylation and the phosphorylation-dependent ubiquitin-proteasome degradation pathway in various mammalian cells [23].

The mitochondria-mediated caspase activation pathway is a major apoptotic pathway characterized by mitochondrial outer membrane permeabilization [24], and degradation of β-catenin by caspase-3 in the signaling pathway of apoptosis is another major contributing factor to tissue and cell damage, including hyperpermeability of endothelial cells [25–28]. We reported previously that IAV infection induces hyperpermeability associated with mitochondrial dysregulation, calcium mobilization, and loss of ZO-1 via protease-activated receptor in HUVECs [8] and apoptosis via mitochondrial membrane depolarization in cardiomyoblasts [9]. Upregulated TNF-α also induces mitochondrial dysregulation through increasing mitochondrial O_2_^-^ production and depleting ATP synthesis, decreasing oxygen consumption [10, 29], and increasing [Ca^2+^]_i_ [30].

**Fig. 7:**
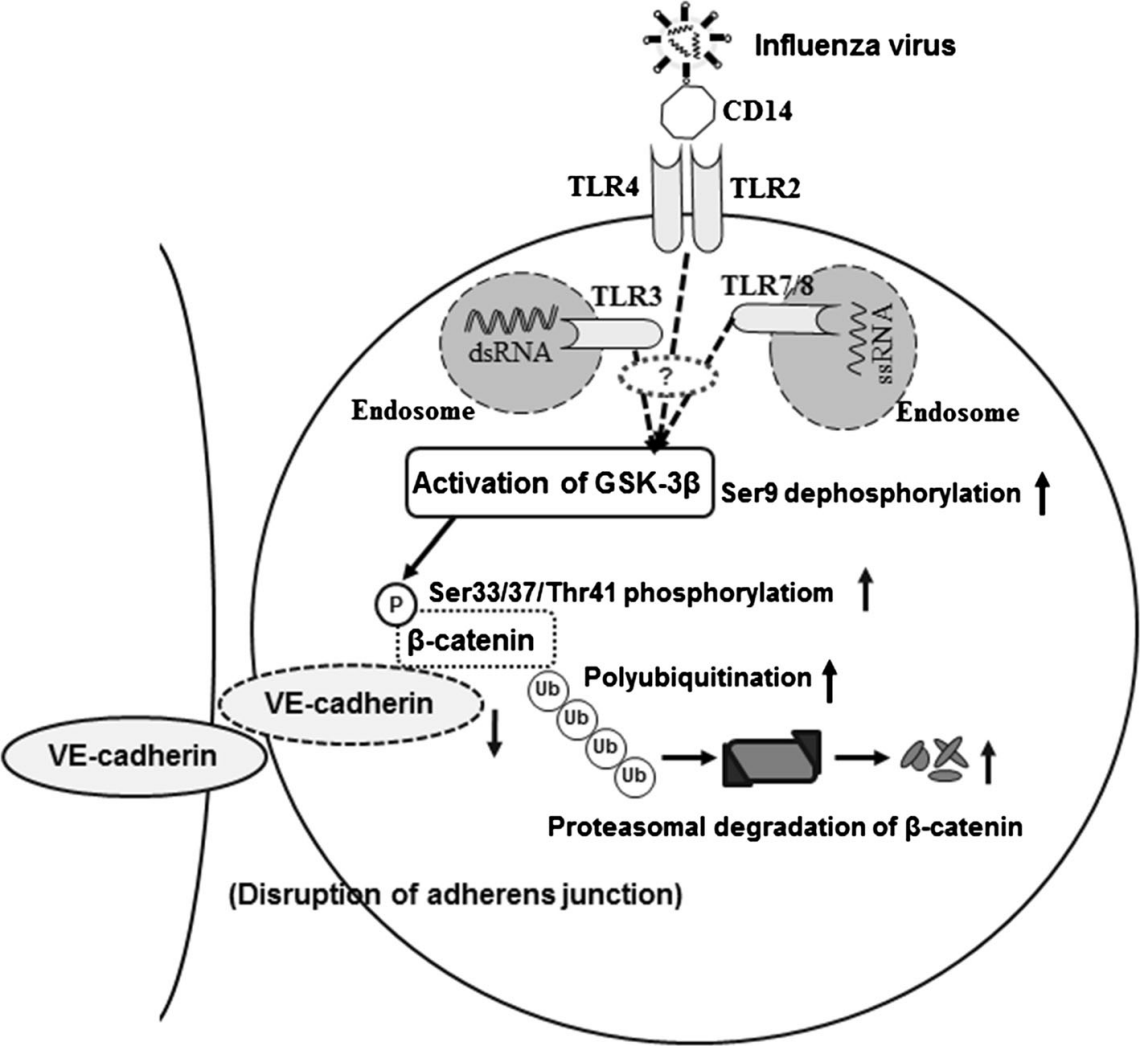
Diagram illustrating the proposed mechanism of disruption of the adherens junctional complexes of vascular endothelial cells after severe IAV infection. Infection of vascular endothelial cells with IAV decreases β-catenin in the VE-cadherin-β-catenin adhesive complex by activation of GSK-3β and the phosphorylation-dependent ubiquitin-proteasome degradation pathway. TLR, Toll-like receptor; dsDNA, double-stranded DNA; ssRNA, single-stranded RNA. The question mark indicates that signal transductions between TLRs and GSK-3β are unidentified

GSK-3β is a serine/threonine protein kinase that has been shown recently to play a key role during the inflammatory response induced by various pathogenic bacteria, such as *Francisella tularensis*, the causative agent of highly virulent tularemia [31], *Burkholderia pseudomallei* [32] and group A streptococcus [33]. Bacterial lipopolysaccharides and lipid A are recognized by the Toll-like receptor (TLR)-2 on the cellular membrane and regulate GSK-3β activity via a phosphoinositide 3-kinase-Akt-dependent pathway [31]. In the present study, we found GSK-3β activation-mediated β-catenin degradation and monolayer hyperpermeability in IAV-infected HUVECs, and we propose a mechanism for the disruption of the adherens junctional complexes of vascular endothelial cells after severe IAV infection (Fig. 7). Since TLR2 and TLR4 bind to viral structural protein with CD14 [34], TLR7/8 binds to ssRNA and TLR3 binds to dsRNA [17], further studies on IAV-induced signal transduction from TLRs to GSK-3β activation are needed. In addition, there is also a need to design new therapeutic agents that can upregulate β-catenin and inhibit GSK-3β in vascular endothelial cells.

In summary, our results show that infection of HUVECs with IAV PR8 markedly decreases β-catenin levels in the VE-cadherin-β-catenin adhesive complex of HUVECs, together with hyperpermeability of HUVEC monolayers. The level of β-catenin in the adhesive complex is tightly regulated by activation of GSK-3β and the phosphorylation-dependent ubiquitin-proteasome degradation pathway. Destruction of the VE-cadherin-β-catenin adhesive complex could be one of the main pathomechanisms of MOF after severe IAV infection.
